# Activation of TNF Receptor 2 Improves Synaptic Plasticity and Enhances Amyloid-β Clearance in an Alzheimer’s Disease Mouse Model with Humanized TNF Receptor 2

**DOI:** 10.3233/JAD-221230

**Published:** 2023-08-01

**Authors:** Natalia Ortí-Casañ, Harald Wajant, H. Bea Kuiperij, Annelien Hooijsma, Leon Tromp, Isabelle L. Poortman, Norick Tadema, Julia H.E. de Lange, Marcel M. Verbeek, Peter P. De Deyn, Petrus J.W. Naudé, Ulrich L.M. Eisel

**Affiliations:** a Department of Molecular Neurobiology, Groningen Institute for Evolutionary Life Sciences, University of Groningen, Groningen, The Netherlands; b Department of Internal Medicine II, University of Würzburg, Würzburg, Germany; c Department of Neurology, Radboud University Medical Center, Donders Institute for Brain, Cognition and Behaviour, Radboud Alzheimer Centre, Nijmegen, The Netherlands; d Department of Laboratory Medicine, Radboud University Medical Center, Nijmegen, The Netherlands; e Department of Neurology and Alzheimer Center, University of Groningen, University Medical Center Groningen, Groningen, The Netherlands

**Keywords:** Alzheimer’s disease, humanized mouse model, neurodegeneration, neuroinflammation, TNF, TNFR2 agonist

## Abstract

**Background:**

Tumor necrosis factor-alpha (TNF-*α*) is a master cytokine involved in a variety of inflammatory and neurological diseases, including Alzheimer’s disease (AD). Therapies that block TNF-*α* proved ineffective as therapeutic for neurodegenerative diseases, which might be explained by the opposing functions of the two receptors of TNF (TNFRs): while TNFR1 stimulation mediates inflammatory and apoptotic pathways, activation of TNFR2 is related to neuroprotection. Despite the success of targeting TNFR2 in a transgenic AD mouse model, research that better mimics the human context is lacking.

**Objective:**

The aim of this study is to investigate whether stimulation of TNFR2 with a TNFR2 agonist is effective in activating human TNFR2 and attenuating AD neuropathology in the J20xhuTNFR2-k/i mouse model.

**Methods:**

Transgenic amyloid-β (Aβ)-overexpressing mice containing a human extracellular TNFR2 domain (J20xhuTNFR2-k/i) were treated with a TNFR2 agonist (NewStar2). After treatment, different behavioral tests and immunohistochemical analysis were performed to assess different parameters, such as cognitive functions, plaque deposition, synaptic plasticity, or microglial phagocytosis.

**Results:**

Treatment with NewStar2 in J20xhuTNFR2-k/i mice resulted in a drastic decrease in plaque load and beta-secretase 1 (BACE-1) compared to controls. Moreover, TNFR2 stimulation increased microglial phagocytic activity, leading to enhanced Aβ clearance. Finally, activation of TNFR2 rescued cognitive impairments and improved synaptic plasticity.

**Conclusion:**

Our findings demonstrate that activation of human TNFR2 ameliorates neuropathology and improves cognitive functions in an AD mouse model. Moreover, our study confirms that the J20xhuTNFR2-k/i mouse model is suitable for testing human TNFR2-specific compounds.

## INTRODUCTION

Alzheimer’s disease (AD) is a neurodegenerative disease and the most common form of dementia that currently affects 50 million people worldwide and for which there is no disease modifying treatment available. Even though the major hallmarks of AD are characterized by synaptic loss and the presence of amyloid-β (Aβ) plaques and neurofibrillary tangles, treatment of AD-associated neuroinflammation has more recently arisen as a new promising therapeutic strategy [1]. Tumor necrosis factor alpha (TNF-*α*) is a master pleiotropic cytokine and a key regulator of the innate and adaptive immune system. In particular, elevated levels of TNF-*α* have been linked to different inflammatory and neurodegenerative disorders, such as rheumatoid arthritis, multiple sclerosis, or AD [2]. Furthermore, TNF-*α* and various TNF-engaged signaling pathways have recently been flagged as risk factors for AD pathogenesis in genetic screens [3].

The discovery of the association of deregulated TNF-*α* expression levels with the pathology of different diseases led to the development of anti-TNF-*α* therapies. However, anti-TNF-*α* therapeutic endeavors failed for neurological disorders but were quite successful in the treatment of various autoimmune diseases [4–6]. The therapeutic failure of TNF blockers in some TNF-driven diseases might be explained by the opposing functions of the two TNF receptors (TNFRs), where stimulation of TNF receptor 1 (TNFR1) by soluble (sTNF) or the transmembrane form of TNF-*α* (mTNF) mainly activates cytotoxic and inflammatory pathways whereas activation of TNF receptor 2 (TNFR2) by mTNF is related to neuroprotective functions, such as immune regulation and tissue regeneration [4, 7, 8]. Indeed, several studies have selectively targeted TNFR1 with inhibitory molecules and/or TNFR2 with agonists and obtained promising results in different neurodegenerative disease models [9–15].

We have recently shown that selective targeting of TNFR2 with the TNFR2 agonist NewStar2 improves AD neuropathology by drastically decreasing Aβ plaque load and enhancing cognition in the J20 AD mouse model [16, 17]. Moreover, we observed that NewStar2 modifies the inflammatory profile of glial cells towards a phagocytic status, essential for Aβ clearance, and decreases expression levels of beta-secretase 1 (BACE-1), partly responsible for the production of Aβ peptides [17]. Thus, our previous study demonstrated that stimulation of TNFR2 is effective in ameliorating neuropathology in an AD mouse model. However, translation of this effect into the human context remains challenging due to differences in the homology of TNFRs between mice and humans. To this end, in the present study, we employed the J20 AD mouse model crossbred with a transgenic humanized mouse model, where the endogenous TNFR2 is exchanged for a chimeric human extracellular TNFR2 domain (J20xhuTNFR2-k/i) [14]. Therefore, TNFR2 in the J20xhuTNFR2-k/i model can exclusively be activated by human TNFR2-directed compounds. Moreover, it has previously been demonstrated that NewStar2 can activate human TNFR2 [16].

The aim of the present study is to investigate and validate whether stimulation of TNFR2 by NewStar2 is effective in activating human TNFR2 and attenuating AD neuropathology in the J20xhuTNFR2-k/i mouse model. Even though the model used in our study is still a mouse model, due to the use of humanized TNFR2, results from this research and the employment of human-directed compounds may shed new light on the potential of targeting human TNFR2 and facilitate the transition to clinical research.

## MATERIAL AND METHODS

### TNFR2 activation

Activation of TNFR2 was achieved by using the TNFR2 agonist NewStar2, previously described by Vargas et al. [16]. Briefly, NewStar2, also known as irrIgG1(N297A)-HC:sc(mu)TNF80 is based on its predecessor Star2 (TNC-sc(mu)TNF80), a fusion protein composed of a short trimerization domain derived of tenascin-C and a sc(mu)TNF80 domain, a domain consisting of three protomers of soluble murine TNF connected by short linkers and carrying two point mutations providing specificity for TNFR2 [18]. Thus, Star2 contains three physically linked soluble TNF trimer domains. In NewStar2, the sc(mu)TNF80 domain has been fused to the C-terminus of the heavy chain of an irrelevant IgG1 molecule. In comparison to Star2, NewStar2 only has two physically linked TNF trimer domains (sc(mu)TNF80). Nevertheless, it is *in vitro* comparable active as Star2 but showed increased serum retention and thus enhanced *in vivo* activity [16].

### Mice

All animal experiments performed in this study were approved by the animal ethics committee of the University of Groningen (AVD1050020186146). To validate the neuroprotective effect of NewStar2 in humanized mice, we employed the J20xhuTNFR2-k/i model. J20 mice overexpress human amyloid-β protein precursor with the Swedish and Indiana mutations and show Aβ deposits at around 5 months of age [19]. J20 mice were initially obtained from the Mutant Mouse Resource and Research Center (MMRRC stock no. 034836-JAX; former JAX stock no. 006293) and were thereafter bred in the animal facility of the University of Groningen. The huTNFR2-k/i transgenic mice have been described previously [14]. Basically, the endogenous TNFR2 is replaced for a chimeric TNFR2 consisting of an extracellular human TNFR2 domain fused to the transmembrane and intracellular mouse TNFR2 domains. J20 and huTNFR2-k/i mice were crossbred to generate the J20xhuTNFR2-k/i line. J20xhuTNFR2-k/i mice were hemizygous for the J20 transgene and homozygous for the huTNFR2-k/i transgene. Mice had access to food and water *ad libitum* and were on a 12 : 12 light/dark cycle.

### Administration and dosage of NewStar2

NewStar2 (2.5 mg/kg in 200μl PBS) or PBS (200μl) as a control was administered twice a week for a period of 6 weeks to a total of 30 male mice starting at 6 months of age via IP injections (NewStar2: *n* = 15; PBS: *n* = 15).

### Behavioral tests

To assess cognitive functions after treatment with NewStar2, mice were subjected to different behavioral tests in the subsequent order: Elevated plus-maze (EPM), Y-maze spontaneous alternation, and Morris water maze (MWM). All tests were performed by the same researcher, who was blinded for the received treatment.

### Habituation and handling

Before commencing with the first behavioral test, mice were habituated to the experimental room and handling by the researcher. Mice were handled separately for 2 min each during 5 consecutive days in the experimental room.

### Elevated plus-maze (EPM)

To assess anxiety-like behavior, mice were tested in an EPM [20]. The maze consisted of two open arms and two closed arms (5.5 cm width × 30 cm length) positioned in a plus shape and elevated 60 cm from the ground. Light intensity was set to  12 lux in the open arms and  10 lux in the center of the maze. Mice were placed in the center of the maze and allowed to freely explore the maze for a duration of 8 minutes. Percentage of time spent in center, open and closed arms was determined using EthoVision XT 11.5 system.

### Y-maze spontaneous alternation

To evaluate short-term spatial memory, mice were tested in a Y-maze to measure spontaneous arm alternation [21]. The maze consisted of three arms (8 cm width × 40 cm length) separated at a 120° angle from each other. Light intensity was adjusted to  10 lux in the center of the maze. Mice were placed in the center of the maze and allowed to freely explore the three arms of the maze for 10 min. Percentage of spontaneous alternation was calculated manually as follows: number of triads / (total number of entries –2) x 100. Triads were defined as three consecutive entries into all three different arms.

### Morris water maze

To investigate hippocampus-dependent spatial memory, mice were tested in the MWM test [22]. The MWM consisted of a circular pool of 135 cm of diameter filled with water. Water temperature was monitored daily and maintained at 24±1°C throughout the test. In order to make the platform invisible to the mice, water-based non-toxic white paint (Dulux Roll-it easy, Belgium) was added to the water. Light intensity was set to  40 lux in the center of the maze and different visual cues were placed on the walls of the experimental room. The MWM had a duration of 10 days, which were divided in 8 days of a training phase and two days of a probe trial phase. Each day of the training phase consisted of 4 trials of maximum 120 s swimming per trial, where mice were trained to find the fixed position of the platform (15 cm of diameter). Time (seconds) necessary to find the platform (escape latency) throughout the 8 days of training was scored using EthoVision XT 11.5 system. After the training phase, a probe trial phase of 2 days (24 and 48 h post-training) was performed. In this phase, the platform was removed from the MWM pool and mice conducted one trial of 100 s swimming per day. Number of crossings through the platform position and time spent in the different quadrants of the pool were scored using EthoVision XT 11.5 system.

### Perfusion and post-fixation

Once all behavioral tests were completed, brain, blood, and cerebrospinal fluid (CSF) were collected from each animal. Mice were terminally anesthetized using 20% sodium pentobarbital and transcardially perfused with 4% paraformaldehyde (PFA)+0.9% heparin saline. Next, brains were post-fixated for 24 h in 4% PFA and cryo-protected with 30% sucrose. Following, brains were frozen and cut into 20μm-thick coronal sections using a cryostat at –20°C.

### Immunohistochemistry for detection of Aβ, tau hyperphosphorylation, Iba1, and GFAP

Free-floating hippocampal sections were washed 3x5 min in 0.01 M Tris-buffered saline (TBS) and incubated in 0.3% hydrogen peroxide (H_2_O_2_) in 0.01 M TBS for 30 min. Sections were rinsed in 0.01 M TBS and pre-incubated in 3% bovine serum albumin (BSA), 0.1% Triton X-100 in 0.01 M TBS (blocking buffer) for 1 h. Subsequently, sections were incubated with primary antibodies mouse anti-Aβ_1 - 16_ (1 : 2000, 6e10, BioLegend), mouse anti-glial fibrillary acidic protein (GFAP) (1 : 10000, #G3893, Sigma), mouse anti-AT8 (1 : 1000, #MN1020, ThermoFisher), or rabbit anti-ionized calcium-binding adapter molecule 1 (Iba1) (1 : 2500, #019-19741, Wako Chemicals) at 4°C for 48 (anti-Aβ) or 72 (anti-Iba1, anti-GFAP) h. For the anti-AT8 antibody, sections were first incubated with the primary antibody for 2 h at room temperature followed by 72 h at 4°C. Thereafter, sections were rinsed 6×5 min in 0.01 M TBS and incubated with Biotin-SP-conjugated secondary antibodies (anti-mouse 1 : 500, #115-065-166; anti-rabbit 1 : 500, #111-065-045, Jackson ImmunoResearch Laboratories) for 2 h at room temperature. Next, sections were incubated in avidin-biotin complex (1 : 500, Vectastain Standard ABC kit, Vector Laboratories) for 2 h and the staining was visualized using 3,3’-Diaminobenzidine (DAB) at a concentration of 0.7 mg/ml (SigmaFAST DAB tablets, Sigma). Finally, sections were mounted onto Menzel Superfrost glass microscope slides (Thermo Scientific) using 1% gelatin, air-dried, dehydrated through a series of ethanol to xylol (2×5 min100% ethanol, 1×5 min 70% ethanol/30% xylol, 1×5 min 30% ethanol/70% xylol, 3×5 min 100% xylol) and coverslipped using DPX mounting medium (Sigma). Images were obtained using a Leica DMI6000 B microscope (Leica Microsystems) at a 100x (Aβ, AT8) or 200x (Iba1, GFAP) magnification.

### Immunofluorescence for detection of Aβ+BACE-1, Aβ+CD68, GFAP+Lipocalin-2, and synapsin-1

Free-floating hippocampal sections were washed 3×5 min in 0.01 M TBS, pre-incubated with 3% BSA, 0.1% Triton X-100 in 0.01 M TBS (blocking buffer) for 1 h and incubated with primary antibodies mouse anti-Aβ_1 - 16_ (1 : 2000, 6e10, BioLegend), rabbit anti-BACE-1 (1 : 200, #5606, Cell Signaling), rat anti-CD68 (1 : 1000, #MCA1957GA, Bio-Rad), mouse anti-GFAP (1 : 10000, #G3893, Sigma), rat anti-lipocalin-2 (Lcn-2) (1 : 100, #70287, Abcam), and/or rabbit anti-synapsin-1 (1 : 1000, #AB1543, Millipore) at 4°C for 24 h. The next day, sections were rinsed 6×5 min in 0.01 M TBS and incubated with Alexa Fluor-conjugated secondary antibodies (AF488-conjugated donkey anti-rabbit, 1 : 500, #A21206; AF555-conjugated donkey anti-mouse, 1 : 500, #A32773 and/or AF488-conjugated donkey anti-rat, 1 : 500, #A21208, Invitrogen) for 2 h at room temperature in the dark. Then, sections were rinsed 3×5 min in 0.01 M TBS, mounted onto Menzel Superfrost glass microscope slides (Thermo Scientific) and coverslipped using Mowiol (Sigma) as mounting medium. Fluorescent images were obtained using the Leica DMI6000 B microscope (Leica Microsystems) at a 200xmagnification.

### Quantification of immunohistochemical stainings

Images at 100x or 200x magnification from Aβ, AT8, Iba1, CD68, GFAP, BACE-1, Lcn-2, and synapsin-1 stainings were quantified and analyzed using ImageJ. The area fraction (percentage of coverage) was quantified by automatically dividing positively stained structures from the selected regions of interest by the total area of the image field. For analysis of CD68 + Aβ staining, both the percentage of CD68 coverage and the CD68-positive cells around 6E10-positive Aβ plaques were quantified. The latter was calculated by automatically establishing a region of interest of 20μm around each 6E10-positive plaque and measuring the CD68-positive area fraction. Specific hippocampal regions (cornu ammonis section 1 (CA1), CA3 and dentate gyrus (DG)) were included in the Iba1, CD68, GFAP, and Lcn-2 analysis. For all immunohistochemical stainings, three brain sections per mouse were analyzed.

### Aβ measurement in CSF samples

CSF was collected in polypropylene tubes and stored at –80°C. Since only very small amounts of CSF were collected per mice, five individual CSF samples within each experimental group were pooled and thus three pools per experimental group were obtained. Each pooled sample was diluted ten times before analysis. CSF Aβ_40_ and Aβ_42_ levels were quantified using the Lumipulse chemiluminescent immunoassay (Fujirebio, Gent, Belgium).

### Statistics

Statistical analyses were performed using GraphPad Prism 8.0. Normally distributed data were analyzed by unpaired Student’s *t* test when comparing two groups or Mann-Whitney test when data was not normally distributed. Two-way ANOVA followed by Bonferroni multiple comparison *post-hoc* test was used to analyze multiple groups. All data are shown as mean±standard error of the mean (SEM). All statistical tests performed were two-tailed and statistically significant differences were considered when *p* < 0.05.

## RESULTS

### NewStar2 rescues behavioral deficits in the J20xhuTNFR2-k/i model

**Fig. 1:**
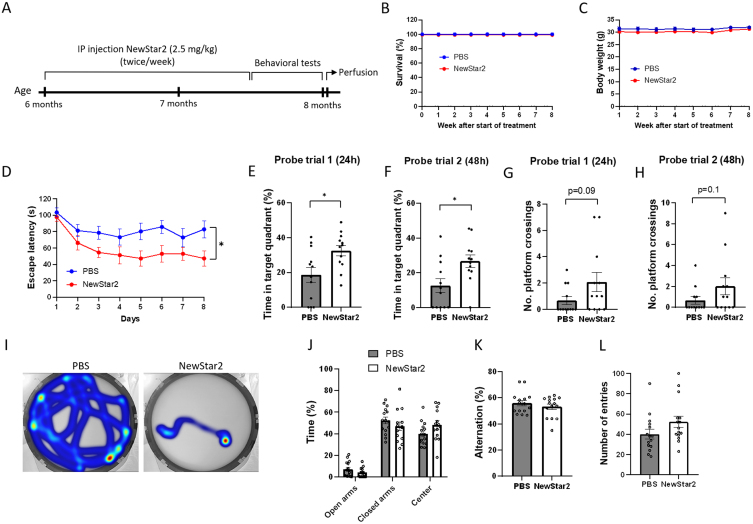
NewStar2 improves cognitive functions. A) Timeline with experimental procedures. Survival (B) and body weight (C) throughout the 8 weeks of experimental procedures (PBS, *n* = 15; NewStar2, *n* = 15). D) Escape latency during the 8 days of training phase in the MWM test (PBS, *n* = 15; NewStar2, *n* = 15; *p* = 0.012; two-way repeated measures ANOVA, Bonferroni post hoc analysis). Time spent in the target quadrant in probe trial 1 (*p* = 0.015) (E) and 2 (*p* = 0.015) (F) (PBS, *n* = 15; NewStar2, *n* = 15, unpaired *t*-test). Number of platform crossings in probe trial 1 (*p* = 0.092) (G) and 2 (*p* = 0.14) (H) (PBS, *n* = 15; NewStar2, *n* = 15, unpaired *t*-test). I) Representative swimming trajectories (heat map) at day 8 of the training phase. Platform was located on the southeast quadrant. J) Time spent in the center, open and closed arms in the EPM test (PBS, *n* = 15; NewStar2, *n* = 15; *p* = 0.92; unpaired *t*-test). Percentage of alternation (*p* = 0.36) (K) and number of total entries (*p* = 0.10) (L) scored in the Y-maze spontaneous alternation (PBS, *n* = 15; NewStar2, *n* = 15, unpaired *t*-test). Data are presented as mean±SEM. *p < 0.05.

To investigate the effects of TNFR2 stimulation on cognitive functions, NewStar2 was administered twice weekly for a period of 6 weeks, and, after the completion of the treatment, different behavioral tests were performed (Fig. 1A). Administration of NewStar2 via IP injections was well tolerated as survival and body weight remained unaffected throughout and after the treatment (Fig. 1B, C). In the MWM test, mice treated with NewStar2 showed a significantly lower escape latency compared to PBS-treated control mice during the training phase (Fig. 1D), which was also observed in their swimming trajectories (Fig. 1I). In the probe trial phase, mice treated with NewStar2 spent significantly more time in the target quadrant (platform quadrant) in both probe trials performed 24 (probe trial 1) and 48 h (probe trial 2) after the training phase, compared to control mice (Fig. 1E, F, respectively). No significant differences were found in the number of platform crossings in probe trials between treated versus control mice, although NewStar2-treated mice presented a tendency to perform more platform crossings compared to control mice (Fig. 1G, H). In the EPM test, no differences in the time spent in open arms, closed arms or center of the maze were found between groups (Fig. 1J). Similarly, in the Y-maze spontaneous alternation test no significant differences were found between groups in the percentage of alternation or the number of total entries performed (Fig. 1K, L).

### NewStar2 improves synaptic plasticity and decreases plaque load and BACE-1 expression levels

**Fig. 2:**
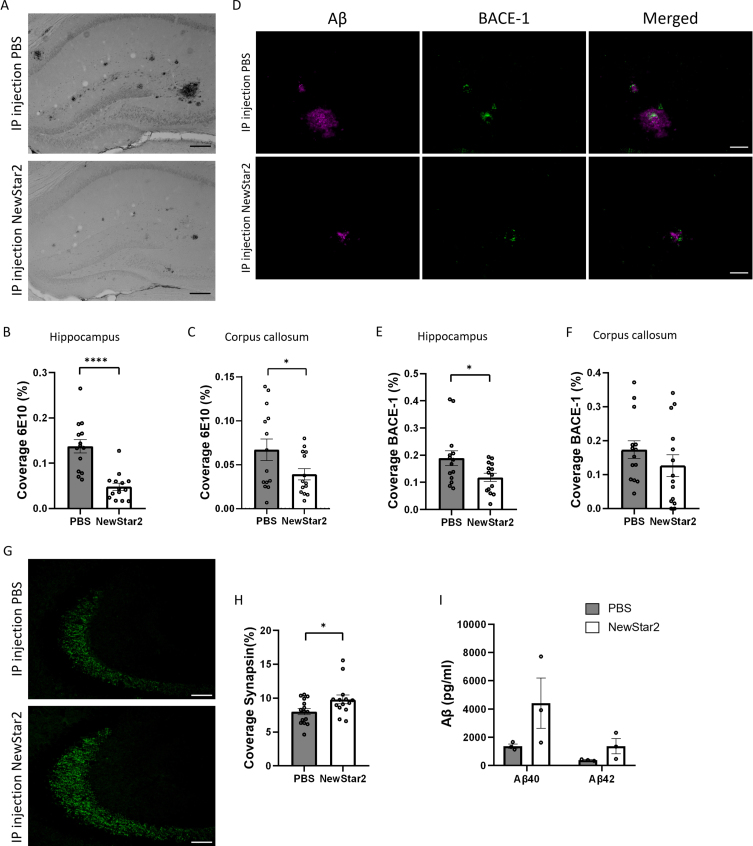
NewStar2 reduces Aβ plaque load and BACE-1 levels, enhances synapsin, and increases Aβ CSF levels. A) Representative hippocampal images of Aβ plaques (6e10) after PBS or NewStar2 administration (Scale bar, 100μm). Quantification of Aβ plaques coverage in hippocampus (B) (PBS, *n* = 14; NewStar2, *n* = 14; *p* < 0.0001; unpaired *t*-test) and corpus callosum (C) (PBS, *n* = 14; NewStar2, *n* = 14; *p* = 0.049; unpaired *t*-test). D) Sections were stained with 6e10 (magenta) for Aβ and anti-BACE-1 (green) for β-secretase BACE-1. Representative images of hippocampus are shown (Scale bar, 100μm). Quantification of BACE-1 coverage in hippocampus (E) (PBS, *n* = 14; NewStar2, *n* = 14; *p* = 0.031; unpaired *t*-test) and corpus callosum (F) (PBS, *n* = 14; NewStar2, *n* = 14; *p* = 0.26; unpaired *t*-test). G) Representative hippocampal images of synapsin 1 (green) after PBS or NewStar2 administration (Scale bar, 100μm). H) Quantification of synapsin 1 coverage in hippocampus (PBS, *n* = 14; NewStar2, *n* = 14; *p* = 0.032; unpaired *t*-test). I) Quantification (pg/ml) of Aβ_40_ and Aβ_42_ levels in CSF (PBS, *n* = 3; NewStar2, *n* = 3; *p* = 0.062; two-way ANOVA, Bonferroni post hoc analysis). Data are presented as mean±SEM. *p < 0.05; ****p < 0.0001.

To determine the effect of NewStar2 on Aβ neuropathology, we assessed the plaque load and BACE-1 expression levels in the brain. Plaque load was significantly decreased in mice treated with NewStar2 compared to control mice in the hippocampus and corpus callosum (Fig. 2A–C). Similarly, NewStar2-treated mice showed a significant reduction in BACE-1 expression levels in the hippocampus compared to PBS-treated controls (Fig. 2D, E). Although not significant, differences in BACE-1 expression levels were observed in the corpus callosum area, where mice treated with NewStar2 showed lower BACE-1 levels compared to control mice (Fig. 2F). Moreover, to investigate the clearance rate of Aβ via CSF pathways, we examined the levels of Aβ_40_ and Aβ_42_ in the CSF of mice. We did not observe significant differences between treatment groups although mice treated with NewStar2 presented a nearly significant trend towards both higher CSF Aβ_40_ and Aβ_42_ compared to PBS-treated mice (Fig. 2I).

Furthermore, we examined the effect of NewStar2 on synaptic plasticity by evaluating the expression levels of synapsin 1, a specific marker of synapses and neurotransmitter release [23]. Administration of NewStar2 resulted in a significant increase in synapsin 1 expression levels in the hippocampus compared to PBS-treated mice (Fig. 2G, H).

Finally, we evaluated whether activation of human TNFR2 by NewStar2 could have an effect on tau phosphorylation by quantifying AT8 levels, a marker for hyperphosphorylated tau filaments [24]. AT8-positive signal in the hippocampus of both NewStar2 and PBS-treated animals was nearly undetectable (Supplementary Figure 1A), showing, therefore, no significant differences between groups (Supplementary Figure 1C). Hippocampal sections from a tau-expressing mouse line (P301L) [25] was employed as positive control (Supplementary Figure 1B).

### NewStar2 stimulates microglial phagocytosis and Aβ uptake

**Fig. 3:**
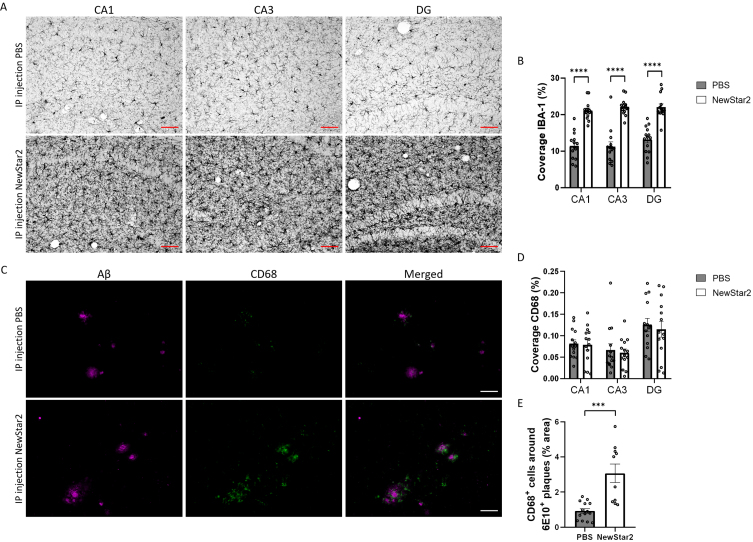
NewStar2 enhances microglial phagocytosis and Aβ clearance. A) Representative images of activated microglia (Iba1) in CA1, CA3 and DG areas after PBS or NewStar2 administration (Scale bar, 100μm). B) Quantification of Iba1 coverage in CA1, CA3, and DG hippocampal areas (PBS, *n* = 14; NewStar2, *n* = 14; *p* < 0.0001; unpaired *t*-test). C) Sections were stained with 6e10 (magenta) for Aβ and anti-CD68 (green) for phagocytic microglia. Representative images of the hippocampus area are shown (Scale bar, 100μm). D) Quantification of CD68 coverage in CA1, CA3, and DG hippocampal areas (PBS, *n* = 14; NewStar2, *n* = 14; *p* = 0.055; two-way ANOVA, Bonferroni post hoc analysis). (E) Quantification of CD68-positive cells around 6e10-positive Aβ plaques (PBS, *n* = 14 mice and *n* = 88 plaques; NewStar2, *n* = 14 mice and *n* = 57 plaques; *p* = 0.0001; unpaired *t*-test). Data are presented as mean±SEM. ***p < 0.001;****p < 0.0001.

To validate the neuroprotective effect of NewStar2, we investigated the inflammatory profile of microglia and their phagocytic activity in different areas of the hippocampus, namely CA1, CA3, and DG. To this end, we evaluated the levels of Iba1, a marker of activated microglia [26] and CD68, a specific marker of phagocytic microglia related to the clearance and degradation of Aβ deposits [27]. Mice treated with NewStar2 showed a drastic increase in Iba1 coverage in all areas of the hippocampus (CA1, CA3, and DG) compared to control mice (Fig. 3A, B). Coverage analysis of CD68 phagocytic-specific microglia revealed no significant differences between groups in CA1, CA3, or DG hippocampal areas (Fig. 3D). Nevertheless, when measuring the area of CD68-positive microglia around individual Aβ plaques, NewStar2-treated mice showed a significant increase in CD68-positive phagocytic microglia around Aβ deposits compared to PBS-treated control mice (Fig. 3C, E).

### Lipocalin-2 expression levels are reduced after NewStar2 administration

**Fig. 4:**
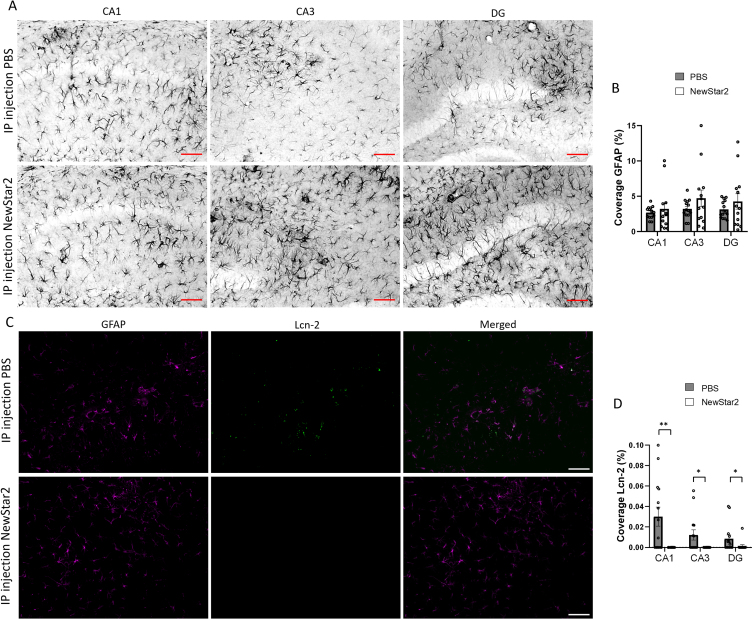
NewStar2 administration contributes to decreasing Lcn-2 expression levels. A) Representative images of activated astrocytes (GFAP) in CA1, CA3, and DG areas after PBS or NewStar2 administration (Scale bar, 100μm). B) Quantification of GFAP coverage in CA1 (*p* = 0.55), CA3 (*p* = 0.21), and DG (*p* = 0.30) hippocampal areas (PBS, *n* = 14; NewStar2, *n* = 14, unpaired *t*-test). C) Sections were stained with anti-GFAP (magenta) for activated astrocytes and anti-Lcn-2 (green). Representative images of the CA1 area are shown (Scale bar, 100μm). D) Quantification of Lcn-2 coverage in CA1 (*p* = 0.0019), CA3 (*p* = 0.015), and DG (*p* = 0.025) hippocampal areas (PBS, *n* = 14; NewStar2, *n* = 14, unpaired *t*-test). Data are presented as mean±SEM. *p < 0.05; **p < 0.01.

To further investigate the involvement of other glial cells after NewStar2 administration, we measured potential changes in astrocytic activity using the GFAP marker as well as the expression levels of Lcn-2, an acute phase protein secreted mainly by astrocytes upon inflammatory stimuli [28]. We observed no significant differences in GFAP coverage between NewStar2 or PBS-treated mice in neither CA1, CA3 nor DG hippocampal areas (Fig. 4A, B). However, NewStar2-treated mice showed a significant decrease in Lcn-2 coverage in all hippocampal areas compared to PBS-treated control mice (Fig. 4C, D).

## DISCUSSION

The neuroprotective role of TNFR2 signaling in neurodegenerative diseases has been extensively demonstrated in different *in vitro* and *in vivo* models [14, 29–32]. In fact, we have recently shown that targeting TNFR2 with the TNFR2-specific agonist NewStar2 improved cognitive functions and ameliorated neuropathology in an AD mouse model [17]. However, stimulation of TNFR2 in clinical studies or research that better mimics the human disease context is lacking. Therefore, in the present study we aimed at validating the role of the murine and human TNFR2 cross-reactive NewStar2 molecule in the J20xhuTNFR2-k/i mouse model, where the chimeric human TNFR2 present in this model permits the employment of human-directed compounds. Our data demonstrate that activation of human TNFR2 by NewStar2 is effective in rescuing cognitive functions and decreasing AD-related neuropathology in the J20xhuTNFR2-k/i mouse model, confirming the usefulness of this model to test human TNFR2-specific agonists.

Firstly, stimulation of TNFR2 via NewStar2 resulted in a significant improvement of synaptic plasticity as indicated by a significant increase in total hippocampal synapsin-1 expression levels. Synaptic dysfunction is a main hallmark in early stages of AD, characterized by a prominent decrease of synapsin-1 in the hippocampus [33], resulting in impaired neurotransmitter release and cognitive impairments [34]. TNF-*α* plays a key role in synaptic homeostasis by regulating the excitability of neurons in response to changes in their electrical activity [35]. However, TNF-*α* signaling through TNFR1 has been related to disturbance of synaptic homeostasis. For instance, increased activation by TNF-*α* of TNFR1 expressed by astrocytes resulted in excessive glutamate release and neuronal dysfunction [35]. This suggests that the generally elevated TNFR1 expression levels found in AD could partly contribute to the reported diminished synaptic plasticity. On the other hand, stimulation of TNFR2 has been associated with neuroprotection and preservation of synaptic homeostasis [36]. Therefore, we hypothesized that activation of TNFR2 by NewStar2 may be able to restore the function of TNF-*α* in maintaining homeostatic synaptic activity, leading to the cognitive improvement observed in our J20xhuTNFR2-k/i mice.

Next to an enhancement of synaptic plasticity, activation of human TNFR2 by NewStar2 led to a robust significant decrease in Aβ plaque load and BACE-1 hippocampal expression levels compared to control mice, BACE-1 being the principal contributor to Aβ_42_ production [37]. Multiple studies have tried inhibiting BACE-1 as strategy to prevent the formation of toxic Aβ peptides. Nevertheless, most clinical trials were unsuccessful or had to be ended due to serious adverse effects [38–41]. The essential neuroprotective role that TNFR2 exerts via the PI3K/Akt pathway is well established [7], and it has been shown that activation of the PI3K/Akt pathway inhibits BACE-1 expression in the APP/PS1 AD mouse model [42]. Thus, the results from the present study indicate that by activating TNFR2, BACE-1 activity could be inhibited, which would lead to the observed reduction in plaque load. These results are consistent with our previous work, where Aβ plaque load and BACE-1 levels were significantly decreased in J20 mice after peripheral administration of NewStar2 [17]. Furthermore, we investigated the clearance of Aβ peptides via CSF pathways. Clearance of Aβ is mainly achieved by phagocytosis of glial cells in the CNS, trans-blood-brain barrier (BBB) transport or CSF absorption [43]. However, multiple studies have demonstrated that, in AD, Aβ clearance via CSF absorption is impaired, resulting in decreased levels of Aβ_42_ in the CSF [44–46]. Consequently, measurement of Aβ levels in the CSF is widely used as a biomarker for AD diagnosis [47]. In our study, we observed that Aβ_40_ and Aβ_42_ CSF levels were increased after NewStar2 administration compared to PBS-treated mice, although the difference was not significant probably due to the small sample size analyzed. This result indicates that activation of TNFR2 might be able to restore CSF absorption, perhaps due to reduced plaque accumulation in brain areas critical for proper CSF absorption functioning. Alternatively, plaque degradation could also lead to increased soluble Aβ in the interstitial fluid and in the CSF.

Besides decreased BACE-1 and increased CSF Aβ levels, activity of glial cells in the CNS might be related to reduced Aβ plaque load in NewStar2-treated mice. Microglia are the resident macrophages in the CNS and, besides protecting the brain from infections and pathogens and maintaining homeostasis, they are crucial for Aβ clearance [48]. However, during AD, dysfunctional microglia are unable to resolve the detected insults, which causes overstimulation of microglia and excessive release of pro-inflammatory cytokines, leading to a vicious cycle of chronic inflammation and cell death [49]. In our study, we first investigated Iba1, a specific marker for activated microglia [26]. We found that activation of TNFR2 via NewStar2 resulted in a drastic increase in Iba1-positive microglia in all areas of the hippocampus compared to PBS-treated mice, indicating that NewStar2 promotes a shift of microglia towards an active state. To further confirm the specific role of microglia after NewStar2 administration, we also examined the levels of CD68, a specific marker of phagocytic microglia. Total levels of CD68-positive microglia in all areas of the hippocampus remained unaltered in NewStar2-treated mice compared to controls, possibly due to a higher amount of Aβ deposits present in the PBS-treated mice. However, analysis of individual Aβ plaques in relation to CD68-positive microglia revealed a significant increase of CD68-positive microglia present in the vicinity of Aβ plaques in mice treated with NewStar2 compared to PBS-treated mice. This result implies that activation of TNFR2 modulates the inflammatory profile of microglia towards a phagocytic state, which increases the uptake and clearance of Aβ deposits, resulting in the observed reduction in plaque load. In support of our study, it has been shown that microglia lacking TNFR2 presented impaired phagocytic activity in the experimental autoimmune encephalomyelitis model [50]. Nevertheless, the precise mechanism by which TNFR2 activation can restore and increase microglial phagocytic activity requires further investigation. In our previous study, we demonstrated that peripheral administration of NewStar2 expands T regulatory cells (Tregs), which also led to an increase in CD68-positive microglia around Aβ plaques [17]. Moreover, a study by Dansokho et al. showed that Tregs ameliorated AD-related pathology and increased plaque-associated microglia in an AD mouse model [51]. Therefore, even though the exact mechanism still needs to be elucidated, we propose that activation of TNFR2 by NewStar2 expands Tregs and increases the phagocytic activity and clearance rate of microglia. Furthermore, we showed in prior experiments that approximately 3% of NewStar2 was able to penetrate the BBB in an *in vitro* transcytosis BBB model [17], indicating that NewStar2 may also cross the BBB *in vivo* through the same mechanisms, although this requires further investigation.

Alongside microglia, astrocytes are another type of glial cells responsible for inflammation and plaque degradation in the CNS [52, 53]. Moreover, under inflammatory conditions, such as in AD, astrocytes secrete an acute-phase protein known as Lcn-2 [54]. A study by Naud $\overset{´}{e}$ et al. demonstrated that Lcn-2 expression is upregulated after TNF-*α* stimulation on neurons, microglia and astrocytes and that, in fact, Lcn-2 production was solely dependent on the TNFR1 signaling pathway [55]. Importantly, the same study showed that Lcn-2 silences TNFR2 by inhibiting the neuroprotective PI3K/Akt pathway, possibly shifting TNF-*α* signaling to a pro-apoptotic pathway [55]. Therefore, in our study, we investigated the effect of stimulating TNFR2 on Lcn-2 levels and astrocytic activation. We found that treatment with NewStar2 significantly decreased Lcn-2 levels in all areas of the hippocampus compared to PBS-treated control mice. Indeed, Lcn-2 levels in NewStar2-treated mice were nearly undetectable in all areas of the hippocampus. These results are in line with other studies that showed very low expression of Lcn-2 in the brain under healthy conditions [55, 56]. Besides, we observed no differences in GFAP levels, a specific marker of activated astrocytes, between groups. This suggests that even though astrocytic activation remains stable after NewStar2 administration, TNFR2 stimulation prevents Lcn-2 production by potentially restoring neuroprotective TNF-*α* signaling pathways. Finally, although Lcn-2 appears to be an attractive therapeutic target for AD therapy, further research regarding its specific function in the brain is needed. For instance, Lcn-2-deficient J20 mice (J20 x Lcn-2 knock-out) showed no improvement in cognitive functions, plaque load or glial activation [28], implying that simply inhibiting Lcn-2 is not enough to attenuate AD-related pathology in this mousemodel.

In addition to investigating Aβ-related neuropathology, we also assessed whether activation of TNFR2 by NewStar2 could ameliorate tau phosphorylation. Accumulation of hyperphosphorylated tau protein and consequent formation of neurofibrillary tangles is one of the main hallmarks of AD [57]. The J20xhuTNFR2-k/i mouse model employed in this study lacks tau pathology and, thus, does not develop neurofibrillary tangles. However, it is plausible that phosphorylated tau filaments are present in our model. Indeed, tau phosphorylation under certain experimental conditions has been previously reported in J20 mice [58, 59]. Our results revealed that tau phosphorylation was nearly absent in both treatment groups and, thus, no significant differences were observed between NewStar2 and PBS-treated mice. This suggests that the quantity of tau phosphorylation in our model was too low to accurately evaluate significant changes after TNFR2 activation. Our data is in line with other studies that demonstrated that J20 mice lack tau hyperphosphorylation [60–62].

Finally, peripheral administration of NewStar2 resulted in an enhancement of cognitive functions compared to PBS-treated mice; specifically, we observed a significant improvement in spatial memory based on the decreased escape latency, increased platform crossings and time spent in the platform quadrant observed in the MWM test. These results can be explained based on the observed increase in synaptic plasticity together with the reduction in plaque load, which may lead to improved neuronal health and cognitive functions. Our data is in line with our previous work, where administration of NewStar2 in the J20 model significantly improved spatial memory [17]. However, data from the spontaneous alternation Y-maze revealed no significant differences in working memory after NewStar2 treatment. Short-term working memory evaluated in the Y-maze is mediated by neurons in the pre-frontal cortex, ventral striatum and hippocampus whereas spatial learning memory assessed in the MWM is mainly hippocampus-dependent [63, 64]. Since the hippocampus is the main brain area affected in the J20xhuTNFR2-k/i mouse model at the age of investigation, these results imply that, while hippocampal dependent-spatial memory is affected, short-term working memory might not be impaired due to the limited involvement of the hippocampus. Our results are supported by a study performed in J20 mice where working memory measured in the spontaneous alternation Y-maze was not impaired compared to wild-type mice [28]. Moreover, NewStar2 was well tolerated throughout the experiment as no differences in anxiety, survivability or body weight were observed. Taken together, the data obtained from the present investigation confirmed that activation of TNFR2 is a potential strategy for AD treatment and that the J20xhuTNFR2-k/i mouse is an appropriate model to test human TNFR2-specific agonists.

Limitations of our study include the utilization of the J20 mouse model, which lacks the presence of neurofibrillary tangles and does therefore not mimic the complete AD context. However, usage of the J20xhuTNFR2-k/i chimeric mouse model allowed us to use a human-specific compound to target the extracellular domain of human TNFR2, giving our results a more translational point of view and suggesting that a modified NewStar2 variant with an irrelevant IgG1 scaffold might also be successful in clinical trials. Nevertheless, testing of NewStar2 in different AD models that express neurofibrillary tangles, such as 3xTg AD mice, should also be considered for future investigations.

Our research is the first *in vivo* study that targeted human TNFR2 with a specific TNFR2 agonist in a humanized AD mouse model. We demonstrated that activation of TNFR2 with NewStar2 improved cognitive functions and resolved AD-related neuropathology in the J20xhuTNFR2-k/i mouse model. In AD, there is a disequilibrium between Aβ production and clearance that contributes to the accumulation of plaque deposition and the development of cognitive impairments present in AD. By activating TNFR2 in our AD mouse model, we observed a decrease in Aβ production based on lower BACE-1 levels together with a remarkable increase in Aβ uptake due to the enhanced phagocytic activity of microglia, which potentially resulted in a drastic reduction in Aβ plaque load and improvement of cognitive functions. Additionally, treatment with NewStar2 improved synaptic plasticity. In conclusion, we could replicate and extend our prior results where we administered NewStar2 to J20 mice [17], which confirms the effectivity of stimulating TNFR2 as potential target for AD. Finally, findings from our study shed new light on the beneficial effects and mechanisms of targeting human TNFR2 with human-specific compounds and the suitability of NewStar2 as potential new therapeutic for AD.

## Supplementary Material

Supplementary Material

## Data Availability

The data supporting the findings of this study are available within the article.
