# Supplementary material for: Activation of TNF Receptor 2 Improves Synaptic Plasticity and Enhances Amyloid-β Clearance in an Alzheimer’s Disease Mouse Model with Humanized TNF Receptor 2
Source: J Alzheimers Dis. 2023 Aug 1;94(3):977–91. doi: 10.3233/JAD-221230 (PMC10578215; doi:10.3233/JAD-221230)
Supplement: Supplementary Material [file jad-94-jad221230-s001.pdf]

Supplementary Material

Activation of TNF Receptor 2 Improves Synaptic Plasticity and Enhances Amyloid-β Clearance in an Alzheimer's Disease Mouse Model with Humanized TNF Receptor 2

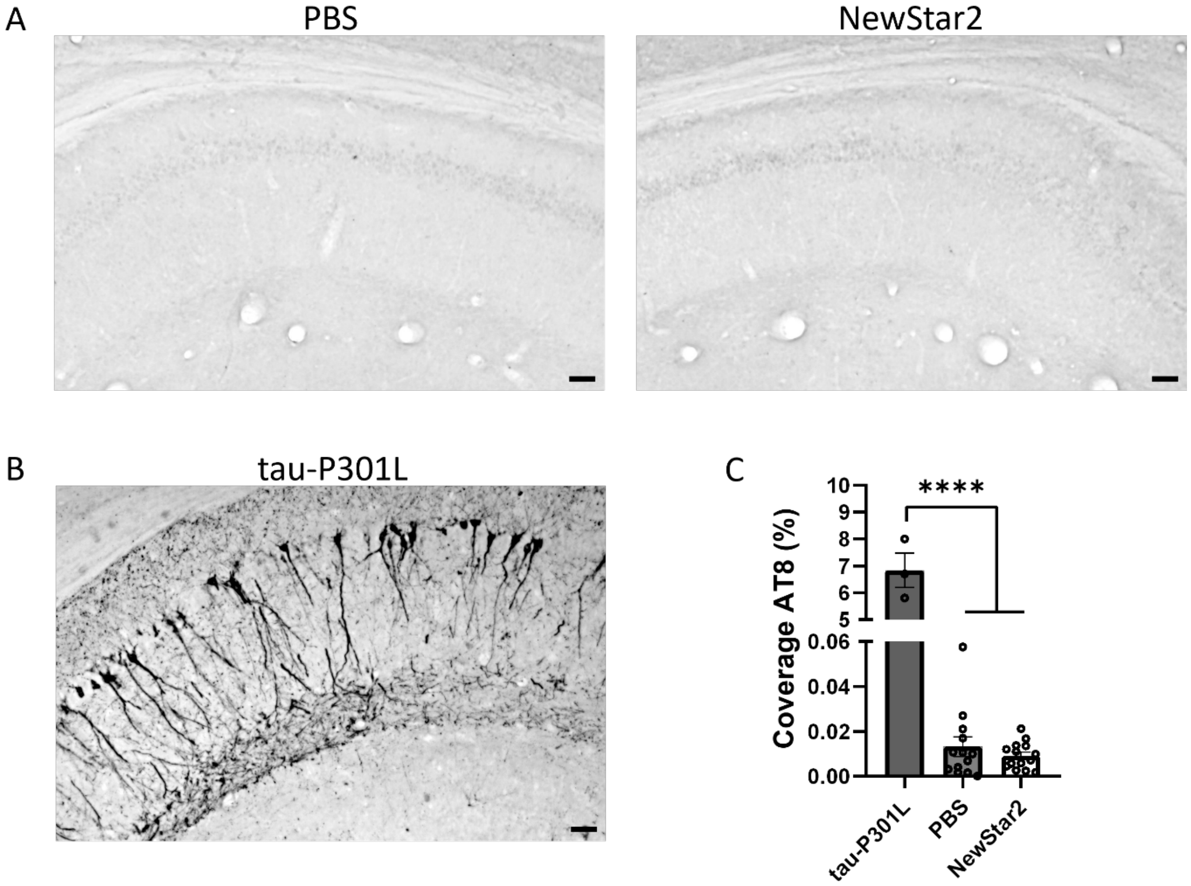

Supplementary Figure 1. Tau phosphorylation is absent in J20xhuTNFR2-k/i mice. A) Representative hippocampal images of tau phosphorylation (AT8) after PBS or NewStar2 administration in J20xhuTNFR2-k/i mice (Scale bar, 50 µm). B) Representative hippocampal image of tau phosphorylation (AT8) in the tau-P301L mouse strain used as positive control (Scale bar, 50 µm). C) Quantification of AT8 coverage in hippocampus (tau-P301L, n=3; PBS, n=13; NewStar2, n=14; $p<0.0001$; one-way ANOVA, Tukey post hoc analysis). Data are presented as mean ± SEM. ****$p<0.0001$.
